# Factors associated with acute clinically important postoperative nausea and vomiting in high-risk patients undergoing laparoscopic gastrointestinal surgery: a secondary analysis of the FDP-PONV trial

**DOI:** 10.3389/fmed.2025.1660659

**Published:** 2025-09-29

**Authors:** Yabin Huang, Jielin Zheng, Ruirui Gong, Yanwei Li, Jiaxin Han, Zhinan Zheng

**Affiliations:** ^1^Department of Anesthesia, The Sixth Affiliated Hospital, Sun Yat-sen University, Guangzhou, Guangdong, China; ^2^Biomedical Innovation Center, The Sixth Affiliated Hospital, Sun Yat-sen University, Guangzhou, Guangdong, China; ^3^Center for Surgery and Anesthesia, The Sixth Affiliated Hospital, Sun Yat-sen University, Guangzhou, Guangdong, China

**Keywords:** postoperative nausea and vomiting, laparoscopic gastrointestinal surgery, influencing factors, prediction model, nomogram

## Abstract

**Background:**

Clinically important postoperative nausea and vomiting (CIPONV) is a highly distressing experience for patients after surgery. CIPONV can occur during the acute phase (0–24 h after surgery) or the delayed phase (25–120 h after surgery), with the acute phase being the primary concern. This study aimed to elucidate the key influencing factors associated with acute CIPONV. This study is based on data from the FDP-PONV trial, which exclusively enrolled patients with 3 or 4 Apfel risk factors. Thus, the findings are specific to this high-risk subgroup.

**Methods:**

Patients participating in the FDP-PONV trial were all included in this study. Acute CIPONV was defined as the occurrence of postoperative nausea and vomiting (PONV) with a simplified PONV impact scale score of 5 or higher during 0–24 h after surgery. The least absolute shrinkage and selection operator was employed to identify the most relevant variables, followed by stepwise regression to refine key factors. A logistic regression model was constructed. Its discrimination was assessed by the receiver operating characteristic curve area under the curve (ROCAUC), and goodness of fit was evaluated via the Hosmer-Lemeshow test and calibration plots.

**Results:**

Among the 1,154 patients, 162 (14.04%) experienced acute CIPONV. Triple prophylactic therapy for PONV, higher preoperative plasma fibrinogen level and higher preoperative monocyte count were negatively associated with acute CIPONV. Motion sickness and/or history of PONV, higher preoperative serum potassium level and the use of quinolones as antibiotic prophylaxis were positively associated with acute CIPONV. Based on these factors, a logistic regression model was constructed. The model showed good discrimination, with ROCAUC of 0.714 (95% confidence interval, 0.675–0.753), an accuracy of 0.621, and good fit (non-significant Hosmer-Lemeshow test, aligned calibration). A nomogram was created for clinical use.

**Conclusion:**

Six factors were identified as key influencing factors for acute CIPONV in high-risk patients undergoing laparoscopic gastrointestinal surgery, which can help clinicians better prevent the occurrence of acute CIPONV in their patients.

## 1 Introduction

In recent years, there has been a growing emphasis on prioritizing patients in healthcare research and evaluation, leading to increased use of patient-reported outcome (PRO) measures that elevate patients’ voices (1). PRO refers to information provided directly by patients about their health status, symptoms, quality of life, or functional abilities without clinician interpretation (1–3). Clinically important postoperative nausea and vomiting (CIPONV) is a crucial PRO proposed by Myles et al. (4), assessing the impact of postoperative nausea and vomiting (PONV) on measuring nausea severity and vomiting episodes using a PONV impact scale. This research indicates that CIPONV affects about 20% of patients experiencing PONV, posing significant concerns in postoperative care (4). It causes discomfort, delayed recovery, longer stays in the post-anesthesia care unit, and increased healthcare costs (5–9). Patients with CIPONV demonstrate lower quality of recovery and a higher likelihood of adverse outcomes compared to those with milder PONV (4).

Furthermore, PONV can manifest within the first 24 h post-surgery (the acute phase) or between 25 to 120 h post-surgery (the delayed phase) (10). Given the complexities of chemotherapy-induced nausea and vomiting, it is evident that the mechanisms at play during the acute phase may differ significantly from those in the delayed phase (11). Consequently, it is imperative to conduct separate analyses for acute and delayed CIPONV to fully understand the distinct pathophysiological processes involved (12). However, the acute phase has traditionally been the focus of concern, with numerous prior studies designating postoperative nausea and vomiting within the first 24 h as the primary endpoint (13–15). Consistent with this approach, The current study also concentrates exclusively on acute CIPONV.

To manage and prevent CIPONV effectively, it is essential to identify the contributing factors. The Apfel score, a prevalent tool for gauging susceptibility to PONV, includes four risk factors: female gender, non-smoking status, a history of PONV or motion sickness, and postoperative opioid use (16). While useful, the Apfel score was developed in 1999 when prophylactic treatments were not standard. Its predictive accuracy, with an area under the receiver operating characteristic curve (ROCAUC) of approximately 0.68, is somewhat limited (17). Factors influencing CIPONV remain poorly defined and require further investigation.

Therefore, the objective of this study was to conduct an analysis of the influencing factors and to develop a predictive model for acute CIPONV, taking into account preoperative conditions and intraoperative data from patients undergoing laparoscopic gastrointestinal surgery. This analysis focuses on patients with 3 or 4 Apfel risk factors from the FDP-PONV trial, aiming to identify determinants of acute CIPONV in this high-risk cohort.

## 2 Materials and methods

### 2.1 Study cohort

The study protocol was approved by the Ethics Committee of the Sixth Affiliated Hospital of Sun Yat-sen University (2024ZSLYEC-201, Chairperson Prof Lin Yao) on April 22, 2024. Given that the study’s retrospective nature relied on existing anonymous data and posed minimal risk to participants, informed consent was formally waived.

This study was a secondary analysis of the data collected in the FDP-PONV trial. The FDP-PONV trial was a randomized, controlled, double-blind clinical study conducted at the Sixth Affiliated Hospital of Sun Yat-sen University from April 2021 to October 2022. The trial was a randomized, double-blind, placebo-controlled study evaluating the efficacy of triple antiemetic prophylaxis (fosaprepitant + dexamethasone + palonosetron) vs. dual prophylaxis (placebo + dexamethasone + palonosetron) (18). This study analyzing the influencing factors for acute CIPONV was conducted from May 30, 2024, to June 15, 2024. The inclusion and exclusion criteria were clearly defined in the published article and represented here. In brief, inclusive criteria were as follows: (a) aged 18–75 years, (b) 3 or 4 Apfel risk factors and (c) scheduled for laparoscopic gastrointestinal surgical procedures under general anesthesia. Exclusion criteria were as follows: (a) American Society of anesthesiologists (ASA) physical status > 3, (b) severe hepatic dysfunction, (c) contraindications to fosaprepitant, 5-HT3 receptor antagonist or dexamethasone, (d) preoperative use of medications with known antiemetic properties, (e) mental disorders or inability to communicate and (f) pregnant women or nursing mothers. All patients participating in the FDP-PONV trial were eligible for this retrospective study, with no further exclusions made to minimize selection bias.

### 2.2 Preoperative medication and anesthetic management

Before the initiation of anesthesia, participants underwent a 30-min intravenous infusion of either fosaprepitant or a placebo solution. During anesthesia induction, all participants received intravenous dexamethasone 5 mg and palonosetron 0.075 mg.

During the administration of general anesthesia, the participants’ vital signs and depth of anesthesia were continuously monitored through electrocardiography, pulse oximetry, capnography, blood pressure measurements, and bispectral index monitoring. The anesthesia was induced and sustained using a method known as total intravenous anesthesia. This involved the controlled infusion of propofol (2–6 μg/mL, Schnider model) and remifentanil (2–6 ng/mL, Minto model) to targeted effect-site concentrations. The adjustment of these concentrations was based on real-time monitoring of the bispectral index and blood pressure. Additionally, muscle relaxation was ensured throughout the procedure with the use of cisatracurium to maintain optimal conditions for anesthesia administration. Upon the conclusion of the surgical procedure, the participants underwent a localized infiltration at the surgical site with 0.5% ropivacaine. The reversal of neuromuscular blockade with neostigmine was determined by the attending anesthesiologist based on clinical assessment of neuromuscular recovery and patient-specific factors. Subsequently, an intravenous patient-controlled analgesia pump, which included hydromorphone, was connected to provide postoperative pain management.

### 2.3 Variables’ definitions

Fifty-five commonly used perioperative clinical features, including baseline characteristics, preoperative conditions, and intraoperative information, were identified as potential predictive factors. These features were collected through an electronic medical record system and case report forms from the FDP-PONV trial. Baseline characteristics and preoperative conditions included age, gender, medical history, ASA physical status classification, the status of anxiety, preoperative laboratory findings, and additional factors. Among these factors, the education level was categorized into three groups: no education or primary school only (low), secondary school (medium), and professional education or university (high) (19). Preoperative sleeping time denoted the total duration of sleep on the night before the surgery. The Fibrinogen Albumin Ratio (FAR) was calculated as plasma fibrinogen concentration (g/L) divided by serum albumin concentration (g/L) (20). Neutrophil-to-Lymphocyte Ratio (NLR) was determined by dividing neutrophil count by lymphocyte count, and Platelet-to-Lymphocyte Ratio (PLR) was calculated by dividing platelet count by lymphocyte count (21). The estimated glomerular filtration rate (eGFR) was derived using the Modification of Diet in Renal Disease (MDRD) equation (22):


$$\begin{array}{c} \text{eGFR}[\mathrm{mL}/\min\cdot 1.73\;m^{2}]=186\times \text{Serum creatinine}\;(\mathrm{mg}/\mathrm{dL})–1.154\\ \times \mathrm{age}\;(\text{years})–0.203\times 0.742\;(\text{if female})\\ \times 1.213\;(\text{if black}) \end{array}$$

Intraoperative information included information such as types of surgery, duration of surgery, concentration of oxygen inhalation during mechanical ventilation, intraoperative anesthetic and analgesic drug dosage, intraoperative use of neostigmine, installation of nasogastric tube, intraoperative hypertension and hypotension, intraoperative fluid balance, types of antibiotic prophylaxis, intraoperative infusion of albumin and the dosage. The duration of pneumoperitoneum referred to the total time of pneumoperitoneum during surgery. Intraoperative hypertension was defined as a mean arterial pressure > 110 mmHg for at least 1 min, while intraoperative hypotension was defined as a mean arterial pressure < 65 mmHg for at least 1 min (18).

### 2.4 Outcome

All patients were classified into either the acute CIPONV group or the non-acute CIPONV group. The simplified PONV impact scale was used to define CIPONV. In the simplified PONV impact scale, nausea intensity or impact was quantified using an ordinal score from 0 to 3, where 0 represents no nausea and 3 represents severe nausea. Vomiting intensity was quantified by the number of vomiting episodes, scored as 0–2 vomits, or 3 if there were three or more vomits. The nausea score and vomiting score were added together to obtain a simplified PONV impact scale score. CIPONV was defined as the occurrence of PONV with the simplified PONV impact scale score of 5 or more (4). The acute phase was defined as the period from 0 to 24 h after surgery, with the starting point specified as the end of surgery.

### 2.5 Statistical analysis

The sample size for a predictive model was calculated using the website https://mvansmeden.shinyapps.io/BeyondEPV/. By setting the number of candidate predictors to 6, the events fraction to 0.14, and the criterion value for rMPSE to 0.03, we determined that a minimum total sample size of 1,080 is required, with a minimally expected event per variable of 25.1 (23).

As the data completeness was 100% for the incidence of PONV within the first 24 h after surgery in the FDP-PONV trial, there was no missing data in this study. Continuous variables following a normal distribution with equal variance were presented as the mean ± standard deviation, with between-group comparisons conducted using the Two Sample t-test. Non-normally distributed continuous variables, variables lacking equal variances, and ranked variables were described using the median (interquartile ranges), with between-group comparisons performed using the Mann–Whitney U test. Categorical variables were depicted using numbers (percentage), and between-group comparisons were carried out using the Pearson Chi-square test or Fisher’s exact test.

In the quest to uncover potential predictive factors associated with CIPONV, we employed the least absolute shrinkage and selection operator (LASSO) to sift through clinically significant variables. Subsequently, stepwise regression was utilized based on the Akaike Information Criterion (AIC) to further refine the selection of the ultimate predictors. We performed 1,000 bootstrap resamples using Bias-Corrected and Accelerated (BCa) confidence intervals to estimate the stability of the results.

Finally, a logistic regression model was developed using the selected factors associated with CIPONV. Model discrimination was assessed via the area under the receiver operating characteristic curve (ROCAUC), while goodness-of-fit was evaluated using the Hosmer-Lemeshow test and calibration plots. To assess the stability of the ROCAUC, 1,000 bootstrap resamples were performed. A nomogram based on the logistic regression model output was developed as a tool for clinical application.

All statistical tests were two-sided, with significance denoted by *p* < 0.05. All analyses were performed using R version 4.2.2. The imputation process was conducted using the “mice” package (version 3.16.0). LASSO was performed using the glmnet package (version 4.1.7). The ROCAUC were produced using the pROC package (version 1.18.5). The nomogram production were conducted using the rms package (version 6.7.1).

## 3 Results

### 3.1 Population clinical characteristics

**Figure 1:**
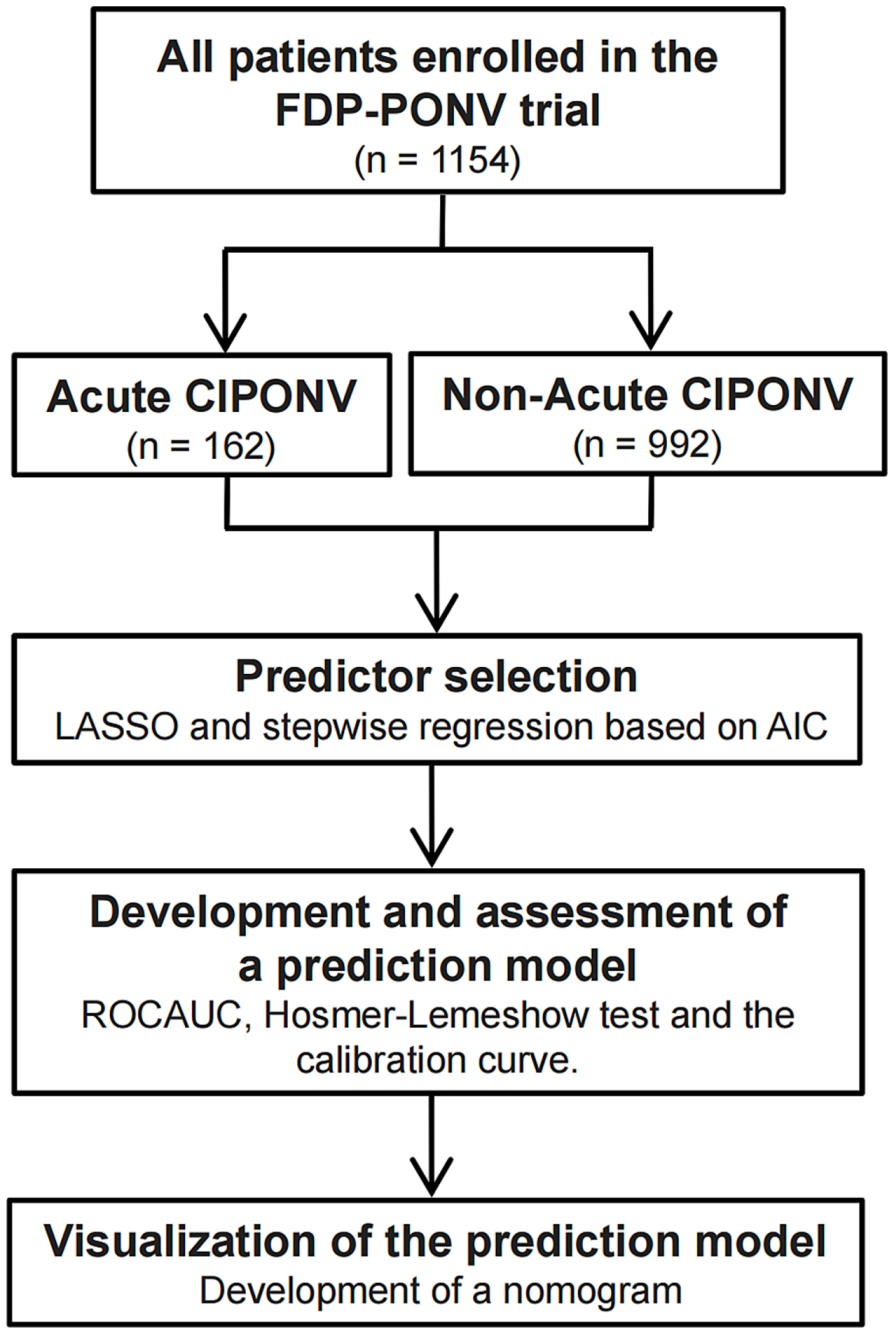
A flow diagram of the study design. CIPONV, clinically important PONV; PONV: postoperative nausea and vomiting; LASSO, least absolute shrinkage and selection operator; AIC, Akaike Information Criterion; ROCAUC, area under the receiver operating characteristic curve.

Figure 1 illustrated the enrollment of all 1,154 patients in the FDP-PONV trial, with no further exclusions. Among them, 162 patients (14.04%) experienced acute CIPONV. A comparison of baseline characteristics and exposure between patients with acute CIPONV and those with non-acute CIPONV is presented in Table 1.

**Table 1:** The baseline characteristics between patients with acute CIPONV and non-acute CIPONV.

Variables	Non-Acute CIPONV 992 (86%) *n* = 992 (86%)	Acute CIPONV n = 162 (14%)	*p*-value
Baseline characteristics			
Sex			0.776
Male	35 (3.5%)	5 (3.1%)	
Female	957 (96.5%)	157 (96.9%)	
Age (year)	56.00 (48.00, 65.00)	54.50 (47.00, 63.75)	0.186
Body mass index (kg m^−2^)	22.07 (20.03, 24.34)	22.04 (19.91, 24.60)	0.851
Education level			0.814
Low	632 (63.7%)	99 (61.1%)	
Medium	181 (18.2%)	32 (19.8%)	
High	179 (18.0%)	31 (19.1%)	
Smoking			>0.999
No	979 (98.7%)	160 (98.8%)	
Yes	13 (1.3%)	2 (1.2%)	
Passive smoking			0.740
No	710 (71.6%)	118 (72.8%)	
Yes	282 (28.4%)	44 (27.2%)	
Motion sickness and/or history of PONV			0.017
No	436 (44.0%)	55 (34.0%)	
Yes	556 (56.0%)	107 (66.0%)	
Apfel score			0.020
3	490 (49.4%)	64 (39.5%)	
4	502 (50.6%)	98 (60.5%)	
Migraine			0.259
No	887 (89.4%)	140 (86.4%)	
Yes	105 (10.6%)	22 (13.6%)	
Hypertension			0.147
No	777 (78.3%)	135 (83.3%)	
Yes	215 (21.7%)	27 (16.7%)	
Diabetes mellitus			0.743
No	902 (90.9%)	146 (90.1%)	
Yes	90 (9.1%)	16 (9.9%)	
ASA physical status			0.375
1	36 (3.6%)	3 (1.9%)	
2	906 (91.3%)	153 (94.4%)	
3	50 (5.0%)	6 (3.7%)	
Preoperative conditions			
Preoperative chemotherapy			0.237
No	715 (72.1%)	124 (76.5%)	
Yes	277 (27.9%)	38 (23.5%)	
Number of preoperative chemotherapy cycles	0.00 (0.00, 2.00)	0.00 (0.00, 0.00)	0.388
Preoperative SAS score (standardized)	31.00 (29.00, 35.00)	32.00 (29.00, 35.75)	0.589
APAIS score	10.00 (6.00, 16.00)	11.00 (6.00, 18.00)	0.126
Preoperative sleeping time (hour)	5 (3, 6)	5 (3, 6)	0.751
Preoperative laboratory test			
Fasting blood glucose (mmol L^−1^)	4.97 (4.56, 5.55)	5.00 (4.60, 5.65)	0.496
Plasma fibrinogen (g L^−1^)	3.10 (2.70, 3.50)	3.00 (2.60, 3.30)	0.033
Serum albumin (g L^−1^)	38.01 (35.85, 40.16)	38.00 (35.56, 40.44)	0.898
FAR	0.08 (0.07, 0.09)	0.08 (0.07, 0.09)	0.070
Serum ALT (U L^−1^)	14.18 (10.34, 21.90)	13.46 (11.10, 20.14)	0.999
Serum AST (U L^−1^)	18.44 (15.18, 24.25)	18.45 (15.59, 23.81)	0.871
Hemoglobin (g L^−1^)	117.00 (103.00, 128.00)	118.00 (104.00, 126.00)	0.541
Serum Na (mmol L^−1^)	141.03 (139.49, 142.45)	141.32 (140.05, 142.72)	0.180
Serum K (mmol L^−1^)	3.85 ± 0.34	3.93 ± 0.34	0.009
Serum Ca (mmol L^−1^)	2.25 (2.18, 2.33)	2.25 (2.18, 2.33)	0.774
Lymphocyte count (×10^9^ L^−1^)	1.61 (1.22, 2.03)	1.57 (1.25, 2.04)	0.864
Monocyte count (×10^9^ L^−1^)	0.45 (0.37, 0.57)	0.43 (0.35, 0.52)	0.017
Neutrophil count (×10^9^ L^−1^)	3.37 (2.54, 4.35)	3.13 (2.48, 4.21)	0.176
Serum inorganic phosphate (mmol L^−1^)	1.23 (1.11, 1.35)	1.25 (1.14, 1.38)	0.203
WBC count (×10^9^ L^−1^)	5.77 (4.62, 6.93)	5.37 (4.58, 6.87)	0.099
RBC count (×10^9^ L^−1^)	4.12 (3.72, 4.46)	4.20 (3.83, 4.50)	0.083
Platelet count (×10^9^ L^−1^)	258.00 (207.00, 321.25)	260.00 (212.75, 310.75)	0.924
NLR	2.01 (1.51, 2.99)	2.14 (1.48, 2.71)	0.555
PLR	162.46 (120.00, 213.58)	167.37 (128.28, 214.03)	0.501
Serum creatinine (μmol L^−1^)	59.54 (52.97, 67.00)	59.15 (53.61, 67.13)	0.964
eGFR [ml (min 1.73m^2^)^−1^]	98.08 (84.52, 112.35)	96.87 (85.12, 112.53)	0.961
Intraoperative information			
Triple prophylactic therapy for PONV			<0.001
No	460 (46.4%)	117 (72.2%)	
Yes	532 (53.6%)	45 (27.8%)	
Types of surgery			0.230
Gastrectomy or small intestinal resection	124 (12.5%)	19 (11.7%)	
Colon resection	437 (44.1%)	85 (52.5%)	
Rectum resection	420 (42.3%)	56 (34.6%)	
Surgery on other site	11 (1.1%)	2 (1.2%)	
Surgery duration (min)	184.00 (145.00, 240.25)	188.00 (141.75, 244.50)	0.944
Inhaled oxygen concentration during mechanical ventilation (%)	45.00 (40.00, 50.00)	45.00 (40.00, 50.00)	0.866
Intraoperative propofol dosage (mg)	1368.00 (1,078.75, 1772.50)	1400.00 (1052.50, 1780.00)	0.641
Intraoperative remifentanil dosage (ug)	1500.0 (1100.0, 2000.0)	1500.0 (1080.0, 2015.0)	0.668
Intraoperative hydromorphone dosage (mg)	1.5 (1.2, 1.5)	1.5 (1.2, 1.5)	0.318
Intraoperative use of neostigmine			0.375
No	362 (36.5%)	65 (40.1%)	
Yes	630 (63.5%)	97 (59.9%)	
Installation of nasogastric tube			0.003
No	875 (88.2%)	129 (79.6%)	
Yes	117 (11.8%)	33 (20.4%)	
Intraoperative hypertension			0.284
No	553 (55.7%)	83 (51.2%)	
Yes	439 (44.3%)	79 (48.8%)	
Intraoperative hypotension			0.541
No	643 (64.8%)	109 (67.3%)	
Yes	349 (35.2%)	53 (32.7%)	
Intravenous infusion fluid volume (mL)	2,450 (2,250, 2,850)	2,450 (2,250, 2,950)	0.820
Blood loss (mL)	50.00 (30.00, 100.00)	50.00 (50.00, 100.00)	0.206
Urine output (mL)	500.00 (300, 800)	500 (250, 800)	0.994
Types of antibiotic prophylaxis			<0.001
Cephalosporins	642 (64.7%)	94 (58.0%)	
Penicillins	212 (21.4%)	23 (14.2%)	
Nitroimidazoles	49 (4.9%)	11 (6.8%)	
Quinolones	89 (9.0%)	34 (21.0%)	
Intraoperative infusion of albumin			0.912
No	951 (95.9%)	155 (95.7%)	
Yes	41 (4.1%)	7 (4.3%)	
Intraoperative albumin dosage (g)	0 (0, 0)	0 (0, 0)	0.923

Data are presented as means ± standard deviations, medians (interquartile ranges) or numbers (percentages) as appropriate. CIPONV, clinically important PONV; PONV, postoperative nausea and vomiting; ASA, American Society of Anesthesiologists; SAS, self-rating anxiety scale; APAIS, Amsterdam preoperative anxiety and information scale; FAR, fibrinogen to albumin ratio; ALT, alanine aminotransferase; AST, aspartate aminotransferase; WBC, white blood cell; RBC, red blood cell; NLR, neutrophil to lymphocyte ratio; PLR, platelet to lymphocyte ratio; eGFR, estimated glomerular filtration rate.

### 3.2 Screening of predictive variables

The LASSO regression was employed to identify the most relevant variables among an initial set of 55 candidate variables. Using the optimal lambda value (*λ* = 0.011691062), the model shrank coefficients of non-informative variables to zero, retaining 16 potential predictive factors with non-zero coefficients (Table 2; Supplementary Figure 1). These 16 variables were then subjected to stepwise regression based on the AIC. Six variables ultimately stood out as independent predictors for acute CIPONV. As shown in Table 3, triple prophylactic therapy for PONV, preoperative plasma fibrinogen level, and preoperative monocyte count were protective factors for acute CIPONV; meanwhile, motion sickness and/or history of PONV, preoperative serum potassium level, and quinolone antibiotic prophylaxis were associated with an increased risk of acute CIPONV. The stability of predictors for acute CIPONV (1,000 bootstrap resamples) is presented in Supplementary Table 1.

**Table 2:** LASSO regression results of potential variables associated with acute CIPONV.

Variables	Coefficient	Lambda. min
(Intercept)	−2.3032	0.011691062
Triple prophylactic therapy for PONV	−0.9208	
ASA physical status II	0.0450	
Motion sickness and/or history of PONV	0.2169	
History of hypertension	−0.0014	
APAIS score	0.0075	
Preoperative plasma fibrinogen level	−0.1859	
Preoperative serum AST level	0.0034	
Preoperative serum K level	0.2691	
Preoperative monocyte count	−0.5229	
Preoperative WBC count	−0.0036	
Preoperative RBC count	0.0310	
Preoperative NLR	−0.0033	
Colon resection	0.1908	
Installation of a nasogastric tube	0.2188	
Types of antibiotic prophylaxis		
Penicillins	−0.0164	
Quinolones	0.7812	

LASSO, least absolute shrinkage and selection operator; CIPONV, clinically important PONV; PONV, postoperative nausea and vomiting; ASA, American Society of Anesthesiologists; APAIS, Amsterdam preoperative anxiety and information scale; AST, aspartate aminotransferase; WBC, white blood cell; RBC, red blood cell; NLR, neutrophil to lymphocyte ratio.

**Table 3:** Final predictors associated with acute CIPONV.

Variables	OR	SE	Z-value	95% CI	*p*-value
Triple prophylactic therapy for PONV					<0.001
No	—	—	—	—	
Yes	0.305	0.193	−6.137	0.209, 0.446	<0.001
Motion sickness and/or history of PONV					0.025
No	—	—	—	—	
Yes	1.515	0.185	2.248	1.055, 2.177	0.025
Preoperative plasma fibrinogen level	0.697^*^	0.148	−2.441	0.522, 0.931	0.015
Preoperative serum K level	1.854^*^	0.262	2.356	1.109, 3.100	0.018
Preoperative monocyte count	0.315^*^	0.582	−1.986	0.101, 0.985	0.047
Types of antibiotic prophylaxis					<0.001
Cephalosporins	—	—	—	—	
Penicillins	0.781	0.252	−0.982	0.476, 1.280	0.326
Nitroimidazoles	1.385	0.366	0.889	0.676, 2.840	0.374
Quinolones	2.877	0.243	4.341	1.786, 4.636	<0.001

CIPONV, clinically important PONV; PONV, postoperative nausea and vomiting; OR, odds ratio; SE, standard error; CI, confidence interval. ^*^The ORs for continuous variables are now explicitly defined as follows: fibrinogen (OR per 1 g/L), monocyte count (OR per 1 × 109 L^−1^), and serum potassium (OR per 1 mmol L^−1^).

### 3.3 Development of a logistic regression model

**Figure 2:**
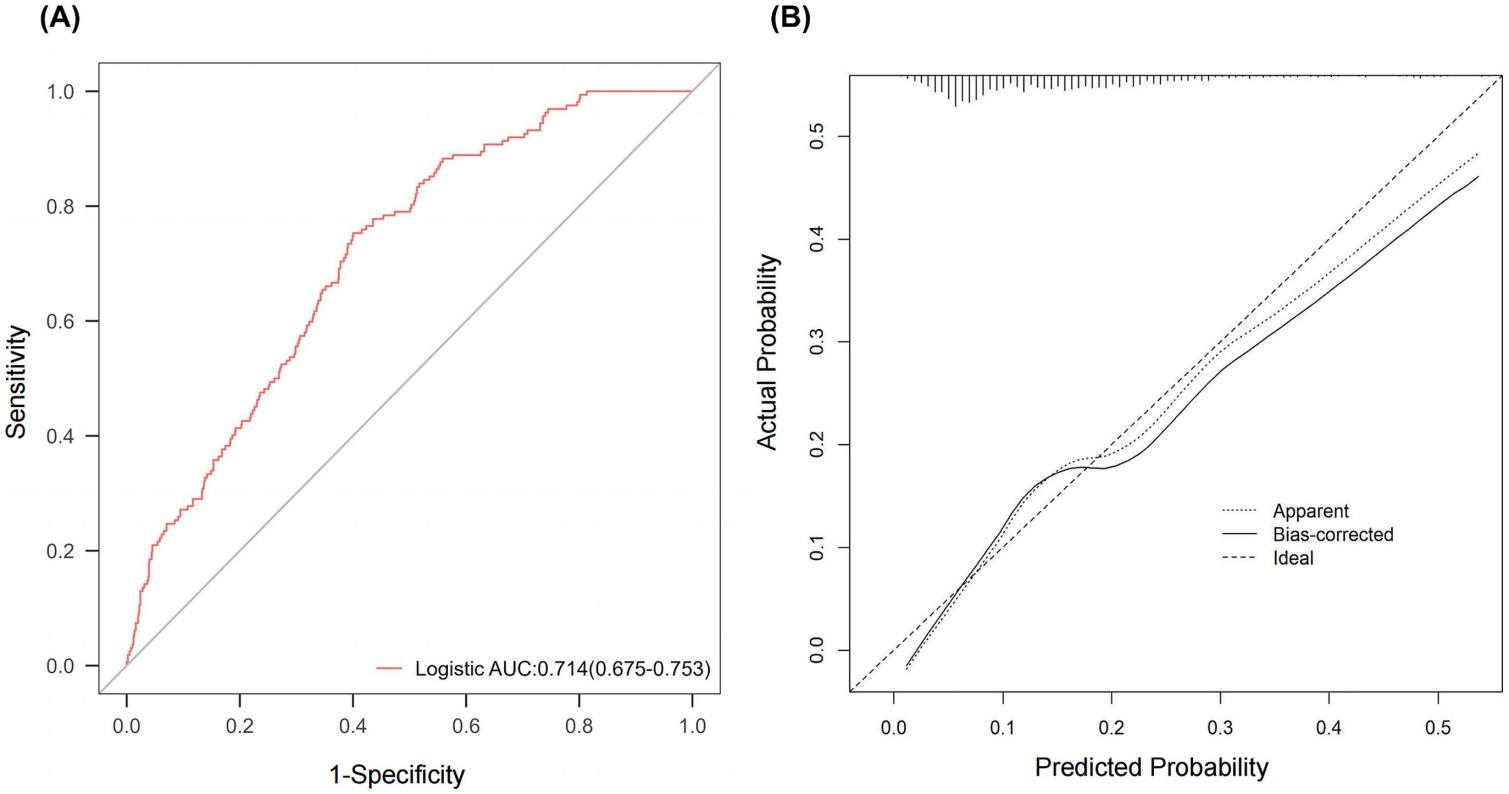
Assessment of the logistic regression prediction model. **(A)** ROC curve. **(B)** Calibration curves. ROC, receiver operating characteristic; AUC, area under the ROC curve; Logistic, logistic regression.

Based on the identified independent influencing factors, a logistic regression model was formulated based on the factors affecting acute CIPONV in patients undergoing laparoscopic gastrointestinal surgery. This model showed an ROCAUC of 0.714 (95% confidence interval, 0.675–0.753, Figure 2A) and an accuracy of 0.621 (95% confidence interval, 0.621–0.622, Table 4). To validate the stability of the ROCAUC, 1,000 bootstrap resamples were performed, which confirmed consistent discriminative performance with a mean ROCAUC of 0.714 (95% bias-corrected and accelerated confidence interval, 0.649–0.739). The Hosmer-Lemeshow test was not statistically significant, indicating the model fits the data well (Table 4). Subsequently, calibration curve analyses were conducted and illustrated a close alignment between the predicted and actual probabilities of occurrence (Figure 2B).

**Table 4:** The logistic regression model performance.

Model	Cut off	ROCAUC (95% CI)	ROCAUC *p*-value	Accuracy (95% CI)	Sensitivity (95% CI)	Specificity (95% CI)	Pos pred value (95% CI)	Neg pred value (95% CI)	HL	HL *p*-value
Logistic	0.134	0.714 (0.675, 0.753)	<0.001	0.621 (0.621, 0.622)	0.753 (0.687, 0.819)	0.600 (0.569, 0.630)	0.235 (0.199, 0.272)	0.937 (0.918, 0.956)	12.951	0.114

ROCAUC, area under the receiver operating characteristic curve; CI, confidence interval; HL, Hosmer-Lemeshow goodness-of-fit test.

**Figure 3:**
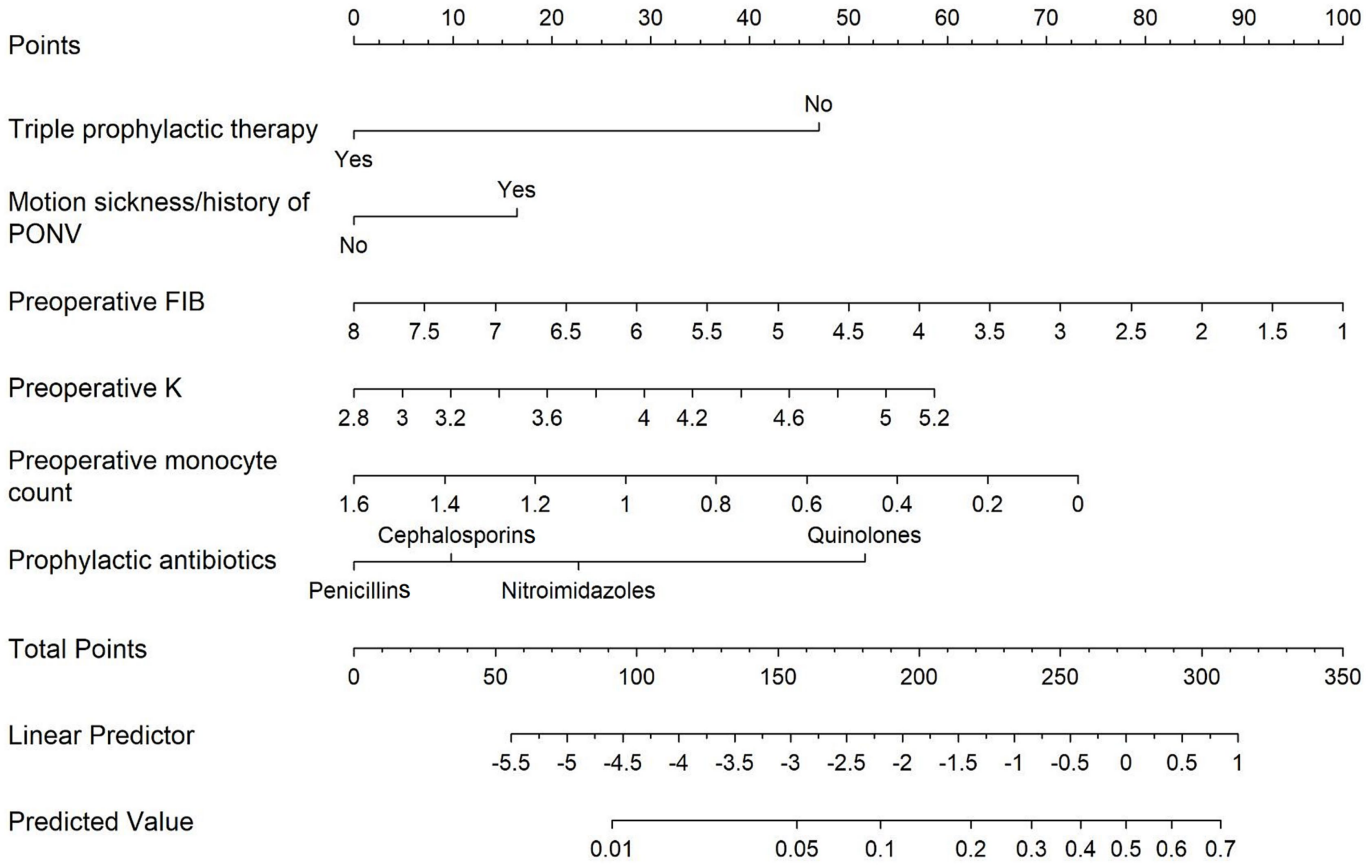
Nomogram for the prediction of acute CIPONV. Each variable axis represents a single node value, and a line is drawn upwards to indicate the corresponding number of points. These numbers are then added up and positioned on the total point axis. Subsequently, a line is drawn downwards to assess the risk of acute CIPONV. CIPONV, clinically important PONV; PONV, postoperative nausea and vomiting; Triple prophylactic therapy, triple prophylactic therapy for PONV; Motion sickness/history of PONV, motion sickness and/or history of PONV; Preoperative FIB, preoperative plasma fibrinogen level; Preoperative K, preoperative serum potassium level.

In order to make the logistic regression model more applicable in clinical situations, a nomogram was developed to aid its practical implementation (Figure 3). The resulting nomogram serves as a visual tool for estimating the likelihood of acute CIPONV based on the aforementioned factors.

## 4 Discussion

The present study identified six key independent factors influencing acute CIPONV in high-risk patients undergoing laparoscopic gastrointestinal surgery—including triple antiemetic prophylaxis, preoperative plasma fibrinogen level, monocyte count, motion sickness/PONV history, serum potassium level, and quinolone use—and developed a predictive model to assess their risk. These findings optimize prophylactic strategies for this specific population (e.g., individualized antibiotic selection and perioperative electrolyte management) and reveal correlations between blood factors (fibrinogen, monocytes, potassium) and CIPONV, providing insights for translational research into PONV pathogenesis and targeted prophylaxis.

PONV is a highly prevalent surgical complication, affecting 30–80% of patients depending on risk factors (16). However, not all PONV episodes are equally impactful from the patient’s perspective. PROs are critical in modern healthcare, ensuring that patient experiences are central to treatment evaluation and intervention design. Among PONV-related PROs, CIPONV is particularly significant, as it quantifies the severity of nausea and vomiting and their functional impact on patients’ daily lives using validated scales (4).

Our analysis identified six predictors of acute CIPONV. When compared with traditional PONV assessment systems, such as the Apfel score and Koivuranta score (16, 24), motion sickness and/or prior PONV history emerged as overlapping risk factors, suggesting shared underlying mechanisms—likely including genetic predispositions that enhance susceptibility to both conditions (25). Notably, the Koivuranta score identifies longer surgical duration as a PONV risk factor, a relationship often attributed to the dose-dependent emetogenic effects of anesthetic agents (26). In contrast, surgical duration and anesthetic factors were not selected as predictors of acute CIPONV in our study. This discrepancy may be explained by the broader physiological perturbations induced by surgical stress, which disrupts the release of inflammatory cytokines (e.g., IFN-*γ*, interleukins 6 and 8) implicated in PONV pathogenesis (27–29).

Female gender and non-smoking status are well-established PONV risk factors in traditional models (16). However, due to the inclusion criteria of the FDP-PONV trial (requiring 3–4 Apfel risk factors), our cohort exhibited a marked predominance of female patients (96.5%) and non-smokers (98.7%), which limited our ability to detect these factors as significant predictors of acute CIPONV. Future research should expand the sample to include more diverse gender and smoking status distributions to comprehensively validate these established risk factors.

Beyond traditional models, this study found several novel predictors: triple prophylactic therapy for PONV, quinolone-based antibiotic prophylaxis, and preoperative blood markers (plasma fibrinogen level, monocyte count, serum potassium level). These factors were not included in the Apfel or Koivuranta scores, indicating that integrating biochemical and surgery-specific variables could enhance the accuracy of CIPONV prediction.

One of the key revelations from this study was the effectiveness of a multimodal prophylactic approach. All participants in this study had an Apfel score of 3–4, and the antiemetic regimen was designed to evaluate triple vs. dual prophylaxis. This design aligned with previous studies that advocate for multimodal antiemetic strategies to improve patient outcomes (30, 31); consistent with this, our analysis identified triple prophylaxis as an independent protective factor for acute CIPONV. For patients who develop acute CIPONV despite prophylactic measures, management should prioritize evidence-based rescue strategies. Key principles include selecting antiemetic agents from pharmacological classes distinct from those used in prophylaxis, such as butyrophenones and benzodiazepines, among others. Future research could explore additional regimens based on triple prophylaxis, for instance, the inclusion of olanzapine.

Fluoroquinolones are generally not considered preferred agents for perioperative prophylaxis in gastrointestinal surgery due to concerns about rising bacterial resistance and potential adverse effects. In our cohort, fluoroquinolones accounted for 10.7% of prophylactic regimens. Notably, among patients who developed CIPONV, 21% had received fluoroquinolones. Although fluoroquinolones are not commonly used in clinical practice, their potential impact on CIPONV cannot be ignored, so we still included them in the variable analysis. Among the four perioperative antibiotic types evaluated (cephalosporins, penicillins, nitroimidazoles, quinolones), multivariate logistic regression showed that quinolones were independently linked to a 2.88-fold increased risk of acute CIPONV. This specificity may stem from quinolones’ unique mechanisms, including gut microbiota disruption, intestinal inflammatory responses, and reduced short-chain fatty acids, which collectively enhance emetogenic susceptibility (32–35). To address potential confounding by infection risk, we included preoperative white blood cell count as a surrogate for baseline inflammatory status. Additionally, our focus on preoperative/intraoperative factors aligns with the acute CIPONV timeline (0–24 h post-surgery), preceding most postoperative infections. However, residual confounding cannot be fully excluded, and future studies should incorporate detailed infection risk stratification (e.g., intraoperative cultures, surgical site infection criteria) to validate this association.

Preoperative blood markers also provided critical insights. Fibrinogen, an acute-phase reactant, has been shown to be elevated in inflammatory states (e.g., acute appendicitis, inflammatory bowel disease) (36, 37). The inverse association between preoperative fibrinogen levels and CIPONV risk, while statistically significant, requires cautious interpretation. Fibrinogen’s role in inflammation and hemostasis suggests potential links to PONV pathophysiology, but direct biological mechanisms remain unclear and warrant further investigation.

Notably, this study is the first to report a statistical association between higher preoperative monocyte counts and reduced acute CIPONV risk. While this finding is novel, its biological basis remains speculative. Monocytes may contribute to immune regulation or gut mucosal homeostasis (38–40), but further preclinical and translational studies are required to validate potential mechanisms.

Preoperative serum potassium level was identified as an independent risk factor for acute CIPONV, with mildly elevated potassium levels within the normal physiological range associated with increased risk, a finding that breaks the traditional perception that “normal range equals safety”: conventional clinical practice focuses only on whether serum potassium is outside the normal range (typically 3.5–5.5 mmol/L), whereas this study suggests that subclinical fluctuations within the normal range may be associated with CIPONV risk, which is particularly important for high-risk populations such as those undergoing laparoscopic gastrointestinal surgery, indicating that preoperative electrolyte assessment needs to be more refined rather than merely satisfying “within the normal range”; serum potassium, a key ion for maintaining cell membrane potential, showed that preoperative hyperkalemia correlated with increased CIPONV risk, consistent with prior reports of electrolyte imbalance-related gastrointestinal dysfunction (41–43), and the mild increase in extracellular potassium concentration may align with the mechanism reported in the literature regarding “hyperkalemia”; however, as an observational study, this study did not verify the causal relationship between potassium ions and CIPONV through animal models or cellular experiments, with mechanistic explanations still relying on inferences based on existing physiological knowledge.

Collectively, these biomarkers illustrate systemic inflammation and metabolic perturbations that modulate susceptibility to CIPONV.

A logistic regression model incorporating these predictors demonstrated moderate discriminative ability (ROCAUC: 0.714), comparable to the Apfel score (ROCAUC: 0.68) and within the 0.60–0.80 range reported for PONV models (14, 15, 17, 44, 45). This performance is reasonable given the multifactorial nature of PONV, where biological heterogeneity (e.g., genetic predispositions, inflammatory responses) and overlapping pathophysiological mechanisms inherently limit model accuracy. Notably, Shim et al. who developed a machine learning model for PONV prediction in patients with intravenous patient-controlled analgesia, reported an ROCAUC of ~0.70, further supporting that our results align with contemporary standards in the field (15). Beyond discriminative ability, the model exhibited good calibration, with a non-significant Hosmer-Lemeshow test and close alignment between predicted and observed probabilities in calibration plots, ensuring reliable risk estimation in clinical practice. To facilitate clinical use, a nomogram was developed: clinicians can input patient-specific data (e.g., antibiotic type, fibrinogen level) to calculate individual risk scores, guiding targeted prophylaxis.

This study has several limitations. First, the cohort was derived from the FDP-PONV trial, which strictly included patients with 3 or 4 Apfel risk factors, resulting in extreme demographics (96.5% female, 98.7% non-smokers). Consequently, the model is validated only for this high-risk subgroup and may not generalize to the broader surgical population. Second, the single-center design limits external validity, necessitating multicenter validation in future studies that should also expand sample sizes, include diverse surgical populations, and design mechanistic experiments to validate these associations. Third, the current study relied solely on the simplified PONV impact scale to define CIPONV, which, while validated for quantifying patient-reported symptom severity, remains a subjective measure. Objective clinical outcomes such as rescue antiemetic usage, unplanned hospital readmissions, or post-anesthesia care unit length of stay were not systematically collected in the original FDP-PONV trial design. Integrating such objective metrics in future studies would strengthen the clinical relevance of CIPONV by bridging subjective symptom burden with tangible healthcare resource utilization or intervention requirements.

Six factors were identified as key influences on acute CIPONV in high-risk patients undergoing laparoscopic gastrointestinal surgery. These findings provide both a foundation for optimizing prophylactic strategies in this specific population and a robust basis for translational studies to advance PONV risk stratification and targeted prophylaxis, though further validation in diverse surgical cohorts is required.

## Data Availability

The raw data supporting the conclusions of this article will be made available by the authors, without undue reservation. Requests to access these datasets should be directed to zhengzhn5@mail.sysu.edu.cn.
