# Supplementary material for: Factors associated with acute clinically important postoperative nausea and vomiting in high-risk patients undergoing laparoscopic gastrointestinal surgery: a secondary analysis of the FDP-PONV trial
Source: Front Med (Lausanne). 2025 Sep 29;12:1660659. doi: 10.3389/fmed.2025.1660659 (PMC12515918; doi:10.3389/fmed.2025.1660659)
Supplement: Supplementary file 1 [file Data_Sheet_1.pdf]

Supplementary Material

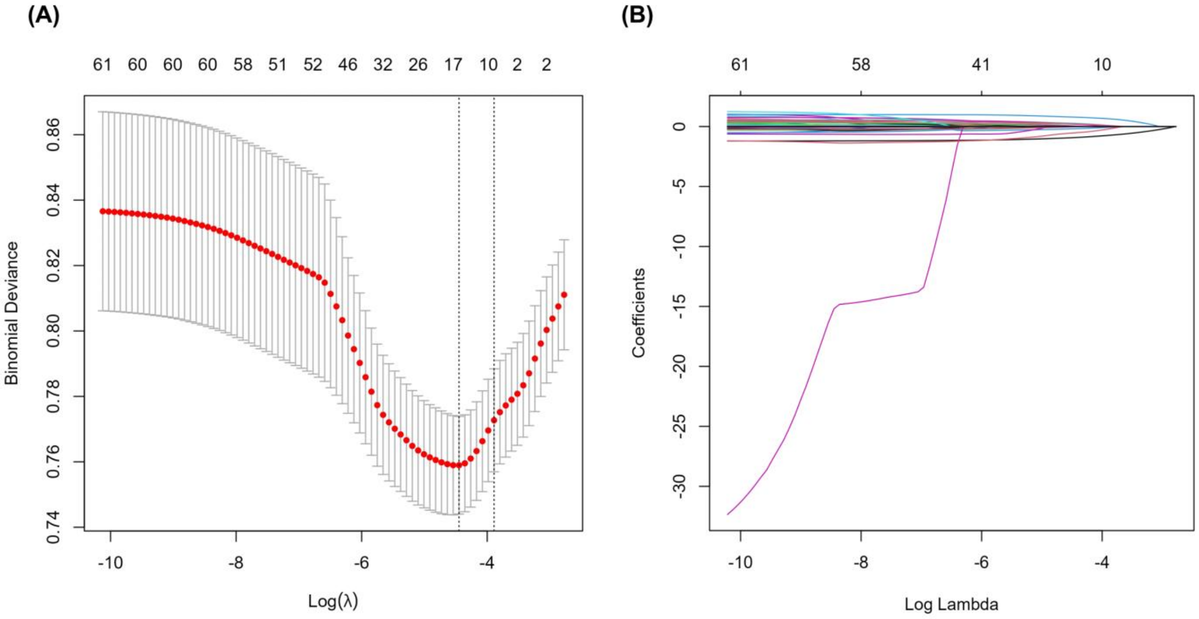

Supplementary Figure 1 Potential predictors selection using LASSO regression.

(A) Parameter selection in the LASSO model used cross-validation via minimum criterion. Partial likelihood deviation (binomial deviation) curves and logarithmic (lambda) curves were plotted. The optimal value was determined using the minimum standard and 1se (1-SE criterion), represented by vertical dashed lines. (B) The coeffcients of each predictor when the 16 potential predictors were included in the LASSO regression model. LASSO: least absolute shrinkage and selection operator; SE: standard error.

Supplementary Table 1 Stability of Predictors for Acute CIPONV: 1000 Bootstrap Resamples with Bias-Corrected and Accelerated Confidence Intervals

Variables	OR	95% bias-corrected and accelerated confidence
(Intercept)	0.087	0.009, 0.803
Triple prophylactic therapy for PONV	0.300	0.203, 0.441
Motion sickness and/or history of PONV	1.514	1.049, 2.169
Preoperative plasma fibrinogen level	0.691	0.516, 0.885
Preoperative serum K level	1.855	1.098, 3.130
Preoperative monocyte count	0.306	0.098, 0.816
Types of antibiotic prophylaxis		
Cephalosporins	—	—
Penicillins	0.797	0.403, 1.174
Nitroimidazoles	1.396	0.552, 2.633
Quinolones	2.865	1.665, 4.546

CIPONV: clinically important PONV; PONV: postoperative nausea and vomiting; OR: odds ratio.
